# PREDICT‐GTN 1: Can we improve the FIGO scoring system in gestational trophoblastic neoplasia?

**DOI:** 10.1002/ijc.34352

**Published:** 2022-12-03

**Authors:** Victoria L. Parker, Matthew C. Winter, John A. Tidy, Barry W. Hancock, Julia E. Palmer, Naveed Sarwar, Baljeet Kaur, Katie McDonald, Xianne Aguiar, Kamaljit Singh, Nick Unsworth, Imran Jabbar, Allan A. Pacey, Robert F. Harrison, Michael J. Seckl

**Affiliations:** ^1^ Department of Oncology and Metabolism The Medical School, The University of Sheffield Sheffield UK; ^2^ Sheffield Centre for Trophoblastic Disease Weston Park Cancer Centre, Sheffield Teaching Hospitals NHS Foundation Trust Sheffield UK; ^3^ Gestational Trophoblastic Disease Centre, Department of Medical Oncology Charing Cross Hospital, Imperial College Healthcare NHS Trust London UK; ^4^ Department of Automatic Control and Systems Engineering The University of Sheffield Sheffield UK

**Keywords:** FIGO, gestational trophoblastic neoplasia, scoring system

## Abstract

Gestational trophoblastic neoplasia (GTN) patients are treated according to the eight‐variable International Federation of Gynaecology and Obstetrics (FIGO) scoring system, that aims to predict first‐line single‐agent chemotherapy resistance. FIGO is imperfect with one‐third of low‐risk patients developing disease resistance to first‐line single‐agent chemotherapy. We aimed to generate simplified models that improve upon FIGO. Logistic regression (LR) and multilayer perceptron (MLP) modelling (n = 4191) generated six models (M1‐6). M1, all eight FIGO variables (scored data); M2, all eight FIGO variables (scored and raw data); M3, nonimaging variables (scored data); M4, nonimaging variables (scored and raw data); M5, imaging variables (scored data); and M6, pretreatment hCG (raw data) + imaging variables (scored data). Performance was compared to FIGO using true and false positive rates, positive and negative predictive values, diagnostic odds ratio, receiver operating characteristic (ROC) curves, Bland‐Altman calibration plots, decision curve analysis and contingency tables. M1‐6 were calibrated and outperformed FIGO on true positive rate and positive predictive value. Using LR and MLP, M1, M2 and M4 generated small improvements to the ROC curve and decision curve analysis. M3, M5 and M6 matched FIGO or performed less well. Compared to FIGO, most (excluding LR M4 and MLP M5) had significant discordance in patient classification (McNemar's test *P* < .05); 55‐112 undertreated, 46‐206 overtreated. Statistical modelling yielded only small gains over FIGO performance, arising through recategorisation of treatment‐resistant patients, with a significant proportion of under/overtreatment as the available data have been used a priori to allocate primary chemotherapy. Streamlining FIGO should now be the focus.

## Graphical abstract

What's new?

The International Federation of Gynaecology and Obstetrics (FIGO) scoring system is used to predict resistance to first‐line single‐agent chemotherapy in gestational trophoblastic neoplasia (GTN) patients. HoweThis system is imperfect, however—one‐third of low‐risk patients develop resistance, and resistance rates increase with FIGO score. Here, six models containing raw and scored data combinations of FIGO variables were analysed in an attempt to improve FIGO scoring for GTN. Analyses show, however, that FIGO cannot be improved by modelling. Any small gains in performance were due to recategorization of treatment‐resistant patients. Future research should focus on optimising existing FIGO scoring strategies for GTN.

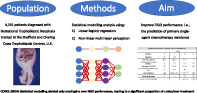

**Abbreviations**
BMP‐9: bone morphogenic protein‐9
CCTDC: Charing Cross Trophoblastic Disease Centre
EMA‐CO: etoposide, methotrexate, actinomycin D/cyclophosphamide and vincristine
FIGO: International Federation of Gynaecolgy and Obstetrics
GTD: gestational trophoblastic disease
GTN: gestational trophoblastic neoplasia
hCG: human chorionic gonadotrophin
IU/L: international units/L
LR: logistic regression
M: model
MEA: methotrexate and etoposide
MLP: multilayer perceptron
n: number
NHS: National Health Service
Pt: threshold probability of administering high‐risk treatment
STDC: Sheffield Trophoblastic Disease Centre

## 1 INTRODUCTION

Gestational trophoblastic disease (GTD) is the umbrella term for a heterogeneous group of placental disorders. These include premalignant complete or partial hydatidiform moles, the malignant subtypes of invasive mole and choriocarcinoma, and the rare placental site‐ and epithelioid trophoblastic tumours. Collectively, the malignant forms of the condition are classified as gestational trophoblastic neoplasia (GTN), which can follow any type of antecedent pregnancy. GTN occurs in 15‐20% of complete hydatidiform mole and 0.5‐1.7% of partial hydatidiform mole cases.^1^, ^2^ GTN is very chemosensitive so most patients are cured of their disease and can proceed to have subsequent, healthy pregnancies.^3^, ^4^

Following a diagnosis of GTN, the International Federation of Gynaecology and Obstetrics scoring system (FIGO) is used to guide primary chemotherapy management. FIGO uses the sum of eight scored variables to determine the risk of primary, single‐agent chemotherapy resistance. Low‐risk patients (total score ≤6) receive first‐line single‐agent chemotherapy, whereas high‐risk patients (total score ≥7) receive first‐line combination treatment, being extremely unlikely to be cured with single‐agent therapy. Currently in the UK first line low‐risk treatment typically involves intramuscular methotrexate, while EMA‐CO (intravenous etoposide, methotrexate, actinomycin D/cyclophosphamide and vincristine) is administered for high‐risk disease.^2^, ^4^

FIGO was developed by a consensus of expert clinicians in 2000, designed to standardise the management of GTN patients. Prior to this, many different scoring systems had been implemented, broadly divided into anatomical, histological and clinical classification systems, yet with little evidence supporting their use. Similarly, FIGO was introduced without prospective validation or in‐depth analysis of the prognostic significance of the eight incorporated risk factors.^5^ It is therefore unsurprising that FIGO is imperfect. Overall, 25‐30% of low‐risk patients are resistant to first‐line single‐agent chemotherapy,^6^, ^7^, ^8^ with rates rising as the FIGO score increases.^9^ Among patients with a total FIGO score of 5 or 6, resistance rates of 70‐80% have been reported,^10^, ^11^ yet more recent UK data suggests these rates to be lower (approximately 40%).^12^ Clinicians have long recognised the need to improve upon FIGO,^2^, ^8^ with the ideal being to refine the prediction prior to commencing chemotherapy. To this avail, published literature has considered novel approaches such as uterine artery pulsatility index either alone^13^ or in combination with circulating angiogenic factors.^14^ Other studies have performed a more detailed interrogation of FIGO variables, including cut offs for pretreatment levels of the hormone human chorionic gonadotrophin (hCG)^11^, ^15^ and the presence of metastatic disease.^12^ Techniques to refine the FIGO prediction have also been proposed following the start of chemotherapy and typically focus upon the hCG response using either hCG half‐life^16^ or computational modelling (nonlinear mixed effects).^17^, ^18^, ^19^

A recent study examined the potential to streamline FIGO into a five‐variable model, simplifying its calculation and reduce scoring variability between centres,^20^ yet there are no published attempts to ‘better’ FIGO performance. In this analysis, using a large dataset of GTN patients derived from the two specialist treatment centres in the United Kingdom, Sheffield (STDC) and Charing Cross Trophoblastic Disease Centres (CCTDC), we explored logistic regression (LR) and multilayer perceptron (MLP) models designed to improve upon FIGO performance.

## 2 METHODS

### 2.1 Data collection

**FIGURE 1:**
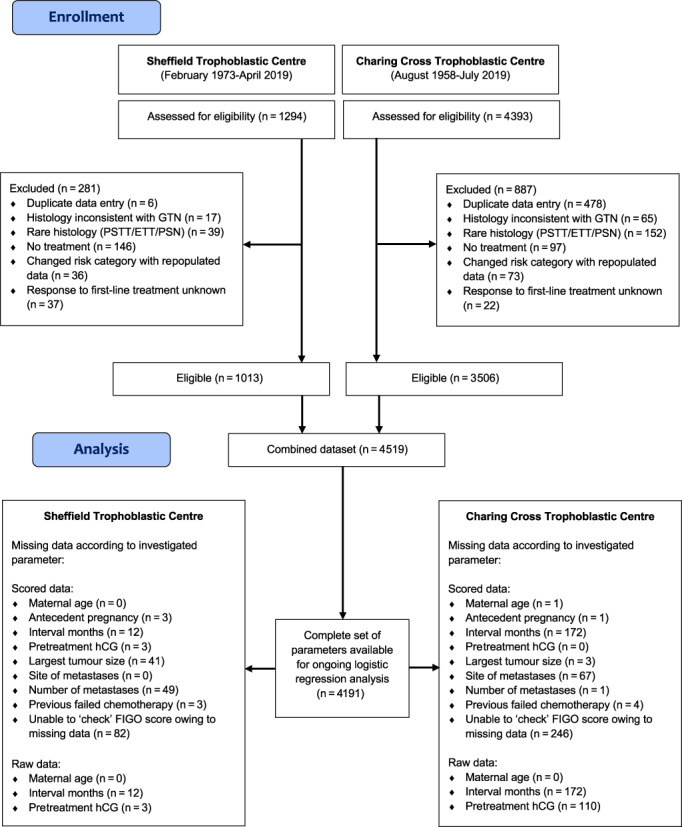
CONSORT diagram. ETT, epithelioid trophoblastic tumour; hCG, human chorionic gonadotrophin; FIGO, International Federation of Gynaecology and Obstetrics; GTN, gestational trophoblastic neoplasia; n, number; PSN, placental site nodule; PSTT, placental site trophoblastic tumour

Patients diagnosed with GTN were identified from the University of Sheffield and National Health Service (NHS) registries of patients maintained by STDC (February 1973‐July 2019) and CCTDC (August 1958‐July 2019) containing 1294 and 4393 patients, respectively. Patients were included if they had: (i) a diagnosis of GTN; (ii) received treatment (chemotherapy or additional surgery) for GTN beyond initial uterine evacuation(s); (iii) a full complement of scored and raw data for the eight prognostic risk‐factors constituting the FIGO score; (iv) details of the primary chemotherapy received; and (v) the response to primary chemotherapy (treatment resistance vs complete response). To investigate an improvement in FIGO performance, all GTN patients (low‐ and high‐risk) were modelled. Treatment resistance to primary chemotherapy was defined as a rise in two or more serial serum hCG levels over 4 weeks, or three or more consecutive hCG readings that did not fall as expected (by approximately 25%) over the same time period.^8^ Patients were excluded if they had: (i) duplicate data entries; (ii) histology inconsistent with a diagnosis of gestational trophoblastic disease following review by expert pathologists; (iii) rare histological subtypes of GTN including placental site‐ or epithelioid trophoblastic tumours and placental‐site nodule; and (iv) a risk category that changed following data cleaning and checking (Figure 1).

The dataset was extensively and independently cleaned and checked by two individuals to ensure complete coverage. This involved identifying and correcting where possible, nonsense (eg, words/inappropriate numbers written in the scored or raw data columns) or human‐error data entries (eg, incorrect score calculated based upon the raw data) and populating missing data. To achieve this, the datasets were cross‐referenced against additional history and treatment information held by the centres. Where discrepancies occurred, the total FIGO score was recalculated using the ‘checked’ data and used in subsequent analyses. Patients whose FIGO risk‐category (low‐ or high‐risk) changed because of data cleaning and checking were excluded from the analysis, as treatment decisions had been made using the ‘original’ data. Scored data was available for all eight risk‐factors. Raw data was available for three parameters: (i) maternal age; (ii) time interval (in months) between the end of the index pregnancy and date of treatment start (defining a month as 28 days); and (iii) pretreatment hCG level.

### 2.2 Diagnosis, treatment and follow‐up

The following criteria were considered indications for commencing chemotherapy for GTN: (i) hCG level > 20 000 IU/L after one or two evacuations; (ii) rising hCG in two consecutive serum samples after one or two evacuations; (iii) hCG plateau in three consecutive serum samples; (iv) heavy vaginal, gastrointestinal or intraperitoneal bleeding; and (v) metastases in the brain, liver or gastrointestinal tract.^21^, ^22^ A raised but declining hCG level 6 months post molar evacuation^23^ or nonmetastatic choriocarcinoma with a normal or falling hCG at the time of diagnosis^24^ were not absolute indications for treatment. Patients were staged according to FIGO 2000 criteria^25^ and their pretreatment histology was centrally reviewed. Patients with low‐risk disease predominantly received a flat‐dose eight‐day methotrexate regimen with alternate day folinic acid. In patients resistant to methotrexate, actinomycin D either alone or in combination with etoposide, or EMA/CO combination chemotherapy were typically administered, depending on the hCG threshold at the development of methotrexate resistance.^26^, ^27^ Since 2012, some patients have received carboplatin as previously described.^8^ For high‐risk patients, methotrexate with etoposide (M‐EA) or EMA/CO have been the preferred first‐line regimens.^10^, ^28^

Serum hCG was measured with a radioimmunoassay using a rabbit polyclonal antibody (CCTDC) or a sandwich chemiluminescent immunometric assay (STDC). Normal hCG levels were defined as <5 IU/L (CCTDC) or <2 IU/L (STDC). Following hCG normalisation, treatment was continued for three further cycles to reduce the risk of relapse.^29^ After completion of treatment, hCG follow‐up varied according to individual centre preference. At the STDC, weekly serum hCG levels are taken for 6 weeks followed by monthly serum and urine hCG for 6 months (STDC). At the CCTDC, 2‐weekly serum and urine hCG levels are required for 6 months, with the frequency decreasing thereafter.^21^, ^22^ Historically, follow‐up continued lifelong, but in 2019 was shortened to 10 years for low‐ and high‐risk patients.^30^

### 2.3 Statistical analysis

Using linear, multivariate LR and nonlinear MLP^31^ analysis (Appendix S1), we explored the prognostic significance of the eight FIGO variables. Based upon the aim to improve FIGO performance with simplified, cost and time efficient models, we selected six models containing raw and scored data combinations of the eight FIGO variables for exploration:

- M1, all variables: maternal age, antecedent pregnancy, interval from antecedent pregnancy, pretreatment hCG, largest tumour size, site of metastases, number of metastases and history of prior failed chemotherapy (scored data);
- M2, all variables: maternal age, antecedent pregnancy, interval from antecedent pregnancy, pretreatment hCG, largest tumour size, site of metastases, number of metastases and history of prior failed chemotherapy (scored and raw data);
- M3, nonimaging variables: maternal age, antecedent pregnancy, interval from antecedent pregnancy, pretreatment hCG and history of prior failed chemotherapy (scored data);
- M4, nonimaging variables: maternal age, antecedent pregnancy, interval from antecedent pregnancy, pretreatment hCG and history of prior failed chemotherapy (scored and raw data);
- M5, imaging variables: largest tumour size, site of metastases, number of metastases (scored data); and
- M6 pretreatment hCG (raw data) + imaging variables: largest tumour size, site of metastases, number of metastases (scored data).

Five‐fold cross validated performance was used to prevent overfitting during model selection. Here, the dataset was randomly divided into five nonoverlapping subgroups of approximately equal sample size and stratified for the prevalence of treatment resistance. Four subgroups were used as a training set and one for validation, repeating the process five times. Each stage of 5‐fold cross validation resulted in a new model estimate.^32^ For LR models, the underlying linear relationship between the log odds of treatment resistance and its predictors allowed the averaging of the five sets of model parameters to produce a single, averaged model. The variability of the parameter estimates about this average was quantified in their coefficient of variation, calculated as the SD divided by the mean for each parameter. A lower coefficient of variation provides greater confidence that the model is representative of the dataset. For MLP models, a single averaged model cannot be generated owing to their inherent nonlinearity, hence the relationship between an averaged model and the 5‐fold cross validated performance breaks down. Instead, a two‐stage strategy was adopted. A subset of the data (n = 3000) was used for 5‐fold cross validation and performance evaluated on a further, ‘hold‐out’, subset (n = 1191). The 5‐fold cross validated procedure produced five operational models and these were applied to the hold‐out data. The mean of the five estimates of log odds of treatment resistance was then used to estimate expected performance.

Model performance was compared to FIGO by fixing the operational value of the false positive rate (FPR) to equal that of FIGO (11.9%). Performance was extensively assessed using a variety of techniques, including conventional measures of true and false positive rates, positive and negative predictive values and the diagnostic odds ratio, where a positive was defined as resistance to primary chemotherapy. Additional measures included receiver operating characteristic and decision curve analyses. The latter compared the net benefit of each model to FIGO and default strategies of treating all patients as high‐risk vs treating all patients as low‐risk over a range of clinically applicable probability thresholds.^33^, ^34^ Net benefit was calculated as: True positive rate × Prevalence − (1‐False positive rate) × (1‐Prevalence) × (Pt/1‐Pt) where Pt was the threshold probability of administering high‐risk treatment.^35^ Contingency tables assessed the discordance between FIGO and the models, detailing the number of patients that would have changed risk category (low‐to‐high‐risk or vice versa) and been over‐ or undertreated as a result of applying the models. Clinically, overtreatment describes the hypothetical administration of primary multiagent chemotherapy to patients who had a complete response to primary single‐agent treatment. Undertreatment involves the hypothetical administration of primary single‐agent treatment to patients who were resistant to multiagent chemotherapy. Calibration of the models was assessed using Bland‐Altman plots,^36^ comparing the performance of the average LR models and the combination MLP models with that of FIGO.

## 3 RESULTS

### 3.1 Demographics

**FIGURE 2:**
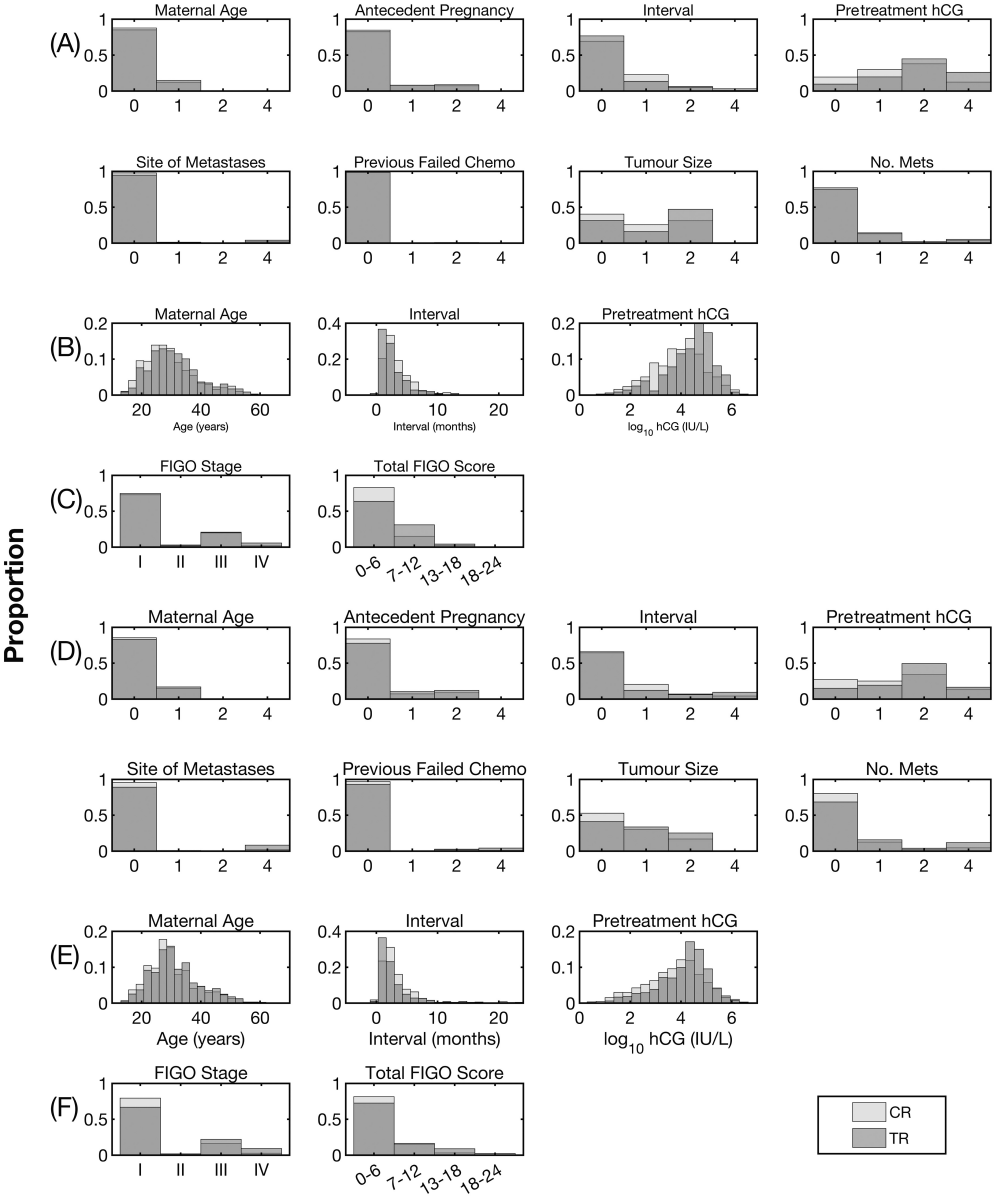
Demographics of the STDC. (A, B, C) and CCTDC (D, E, F) datasets detailing the response to primary chemotherapy. (A) and (D) Scored data for the eight risk‐factors constituting the FIGO. (B) and (E) Raw data for the three FIGO risk‐factors that permit this investigation. (C) and (F) Breakdown of the FIGO stage and total FIGO score. The raw data for interval (B) and (E) is restricted to 24 months for data presentation purposes, with 125 patients (STDC n = 8, CCTDC n = 117) having intervals >24 months (median 42 months, interquartile range 43 [31‐74 months]). CCTDC, Charing Cross Trophoblastic Disease Centre; CR, complete response to primary chemotherapy; hCG, human chorionic gonadotrophin; FIGO, International Federation of Gynaecology and Obstetrics scoring system; STDC, Sheffield Trophoblastic Disease Centre; TR, resistance to primary chemotherapy

The combined dataset contained 4519 patients of which 4191 were eligible for inclusion (Figure 1). Figure 2 details the demographics of the STDC and CCTDC datasets according to the FIGO variables. Tables 1 and S1 describe FIGO performance upon conventional performance measures, revealing the system to operate with a poor sensitivity and positive predictive value, high specificity and moderate negative predictive value. Calibration analysis revealed the difference between observed and predicted deciles of probability to lie within the 95% limits of agreement (Figure S1).

**TABLE 1:** Performance of the FIGO and the six models upon logistic regression and multilayer perceptron analysis

		TPR (%)		FPR (%)		PPV (%)		NPV (%)		DOR	
Model number	Model description	LR	MLP	LR	MLP	LR	MLP	LR	MLP	LR	MLP
	FIGO	17.10	–	11.90	–	44.40	–	65.60	–	1.50	–
M1	All variables (scored data)	21.90	25.40	11.90	11.90	50.50	54.30	67.00	68.00	2.10	2.5
M2	All variables (scored and raw data)	21.80	24.90	11.90	11.90	50.50	53.80	66.90	67.80	2.10	2.5
M3	Nonimaging variables (scored data)	20.00	28.00	11.80	15.80	48.40	49.60	66.40	67.80	1.90	2.1
M4	Nonimaging variables (scored and raw data)	18.80	27.40	11.90	11.90	46.70	56.10	66.10	68.60	1.70	2.8
M5	Imaging variables (scored data)	16.90	18.30	8.90	11.80	51.10	46.20	66.30	66.00	2.10	1.7
M6	log hCG (raw data) + imaging variables (scored data)	19.80	24.50	11.90	11.90	48.00	53.50	66.40	67.70	1.80	2.4

Abbreviations: DOR, diagnostic odds ratio; FIGO, International Federation of Gynaecology and Obstetrics scoring system; FPR, false positive rate; hCG, human chorionic gonadotrophin; log, logarithm; LR, logistic regression; MLP, multilayer perceptron; M, model; NPV, negative predictive value; PPV, positive predictive value; TPR, true positive rate.

### 3.2 Model development

Table 1 details conventional performance parameters, showing FIGO to be outperformed by LR and MLP, particularly in true positive rate and positive predictive value. Across the parameters, MLP model performance was generally slightly superior to LR. Compared to FIGO, MLP improved each parameter by 0.4‐11.7% vs 0.2‐6.7% on LR.

Table 2 summarises the LR coefficients, significance and coefficient of variation for the variables included within each model. The MLP has numerous parameters (101 herein) which are not interpretable in the same way as LR parameters (change in log odds for unit change in variable), so are not useful in themselves.

**TABLE 2:** Breakdown of the logistic regression coefficients, prognostic significance and coefficients of variation for the variables within the six models

		FIGO scored data										Raw data		
Model number	Model description		Constant	Age	Antecedent pregnancy	Interval	Pretreatment hCG	Site of metastases	Previous failed chemotherapy	Largest tumour size	Number of metastases	Age	Interval	Log pretreatment hCG
M1	All variables (scored data)	Coefficient	−1.18	0.09	−0.16	0.07	0.21	0.15	0.34	0.17	0.09	–	–	–
		*P*‐values	0.00	40.74	4.78	9.95	0.00	2.59	0.00	0.13	8.75	–	–	–
		CoV	0.66	17.16	10.68	9.67	2.59	9.06	4.71	5.30	11.44	–	–	–
M2	All variables (scored and raw data)	Coefficient	−2.41	–	−0.16	–	–	0.15	0.38	0.12	0.09	0.02	0.00	0.30
		*P*‐values	0.00	–	6.34	–	–	2.41	0.00	2.62	6.65	0.14	22.45	0.00
		CoV	0.85	–	13.21	–	–	8.97	4.58	6.82	10.56	4.01	21.84	1.85
M3	Nonimaging variables (scored data)	Coefficient	−1.16	0.12	−0.07	0.10	0.29	–	0.37	–	–	–	–	–
		*P*‐values	0.00	25.91	34.66	1.33	0.00	–	0.00	–	–	–	–	–
		CoV	0.60	12.39	19.45	5.33	1.19	–	3.46	–	–	–	–	–
M4	Nonimaging variables (scored and raw data)	Coefficient	−2.59	–	−0.06	–	–	–	0.43	–	–	0.02	0.01	0.37
		*P*‐values	0.00	–	44.97	–	–	–	0.00	–	–	0.06	6.20	0.00
		CoV	0.96	–	33.25	–	–	–	3.26	–	–	3.85	13.37	1.25
M5	Imaging variables (scored data)	Coefficient	−0.90	–	–	–	–	0.18	–	0.28	0.16	–	–	–
		*P*‐values	0.00	–	–	–	–	0.39	–	0.00	0.03	–	–	–
		CoV	0.50	–	–	–	–	6.73	–	2.76	4.29	–	–	–
M6	log hCG (raw data) + imaging variables (scored data)	Coefficient	−1.75	–	–	–	–	0.17	–	0.14	0.12	–	–	0.25
		*P*‐values	0.00	–	–	–	–	0.46	–	1.07	1.00	–	–	0.00
		CoV	1.23					6.79	–	6.23	6.28	–	–	2.50

Abbreviations: CoV, coefficient of variation; FIGO, International Federation of Gynaecology and Obstetrics scoring system; hCG, human chorionic gonadotrophin; log, logarithm; M, model.

### 3.3 M1, all variables (scored data)

Using LR, the area under the curve matched that of FIGO (0.62) (Figure S2A) and improved marginally to 0.63 on MLP (Figure S2D) with an uplift in the ROC curve in the bottom left‐hand corner; the area of interest. Calibration analysis revealed the difference between observed and predicted deciles of probability to lie within the 95% limits of agreement (Figure S2B,E). Decision curve analysis showed a small net benefit at a decision probability threshold equivalent to a total FIGO score of 7. This would translate to 18 (LR) and 21 (MLP hold‐out dataset) patients in 1000 being correctly classified as treatment resistant using the model as opposed to FIGO (Figure S2C,F). Table 3 demonstrates a significant discordance between FIGO and M1 LR (72 patients, McNemar's test *P* = .0002) and MLP analyses (98 patients, *p* ≈ 0). A more detailed analysis of cases that would have changed risk as a result of applying the model, revealed significant over‐ (n = 100 and n = 152) and undertreatment (n = 55 and n = 83) on LR and MLP analysis, respectively (Table 4).

**TABLE 3:** Contingency table comparison of the prediction of FIGO versus models 1 to 6 using logistic regression or multilayer perceptron analysis

	LR			MLP	
	FIGO (n patients)			FIGO (n patients)	
MODEL 1	TR	CR	MODEL 1	TR	CR
**TR**	2473	227	**TR**	1638	333
**CR**	155	1336	**CR**	235	794

MODEL 2	TR	CR	MODEL 2	TR	CR
**TR**	2443	256	**TR**	1720	245
**CR**	185	1307	**CR**	156	879

MODEL 3	TR	CR	MODEL 3	TR	CR
**TR**	2401	272	**TR**	1595	328
**CR**	227	1291	**CR**	278	799

MODEL 4	TR	CR	MODEL 4	TR	CR
**TR**	2380	273	**TR**	1623	369
**CR**	248	1290	**CR**	250	758

MODEL 5	TR	CR	MODEL 5	TR	CR
**TR**	2513	191	**TR**	1683	213
**CR**	115	1372	**CR**	190	914

MODEL 6	TR	CR	MODEL 6	TR	CR
**TR**	2490	178	**TR**	1644	318
**CR**	138	1,385	**CR**	229	809

Abbreviations: CR, complete response to primary chemotherapy; FIGO, International Federation of Gynaecology and Obstetrics scoring system; LR, logistic regression analysis; MLP, multilayer perceptron analysis; M, model; n, number; TR, resistance to primary chemotherapy.

**TABLE 4:** Risk change analysis

	LR					MLP			
	M1 (n patients)					M1 (n patients)			
	TP	TN	FP	FN		TP	TN	FP	FN
Low ‐to high risk	127	0	100	0	Low ‐to high risk	171	0	152	0
High‐ to low‐risk	0	100	0	55	High‐ to low‐risk	0	162	0	83

	M2 (n patients)					M2 (n patients)			
	TP	TN	FP	FN		TP	TN	FP	FN
Low ‐to high risk	135	0	121	0	Low ‐to high risk	134	0	99	0
High‐ to low‐risk	0	121	0	64	High‐ to low‐risk	0	111	0	57

	M3 (n patients)					M3 (n patients)			
	TP	TN	FP	FN		TP	TN	FP	FN
Low ‐to high risk	143	0	127	0	Low ‐to high risk	188	0	206	0
High‐ to low‐risk	0	129	0	100	High‐ to low‐risk	0	140	0	72

	M4 (n patients)					M4 (n patients)			
	TP	TN	FP	FN		TP	TN	FP	FN
Low ‐to high risk	137	0	136	0	Low ‐to high risk	203	0	157	0
High‐ to low‐risk	0	136	0	112	High‐ to low‐risk	0	166	0	93

	M5 (n patients)					M5 (n patients)			
	TP	TN	FP	FN		TP	TN	FP	FN
Low ‐to high risk	61	0	48	0	Low ‐to high risk	91	0	111	0
High‐ to low‐risk	0	130	0	67	High‐ to low‐risk	0	122	0	79

	M6 (n patients)					M6 (n patients)			
	TP	TN	FP	FN		TP	TN	FP	FN
Low ‐to high risk	111	0	67	0	Low ‐to high risk	173	0	135	0
High‐ to low‐risk	0	67	0	71	High‐ to low‐risk	0	145	0	94

*Note*: Outcome of risk‐category changes by applying the models based upon the patients' actual response to primary chemotherapy. FP represents overtreatment and FN represents undertreatment.

Abbreviations: FP, false positive; FN, false negative; LR, logistic regression analysis; MLP, multilayer perceptron analysis; M, model; n, number; TP, true positive; TN, true negative.

### 3.4 M2, all variables (scored and raw data)

FIGO area under the curve was improved upon by both LR (0.64, Figure S3A) and MLP (0.66, Figure S3D), with an uplift in the receiver operating characteristic curve in the bottom left‐hand corner; the area of interest. Calibration analysis revealed the difference between observed and predicted deciles of probability to lie within the 95% limits of agreement (Figure S3B,E). Decision curve analysis showed a small net benefit at a decision probability threshold equivalent to a total FIGO score of 7. This would translate to 19 (LR) and 41 (MLP) patients in 1000 being correctly classified as treatment resistant using the model as opposed to FIGO (Figure S3C,F). Contingency table analysis revealed a discordance of 71 and 89 patients between FIGO and M2 on LR (McNemar's test *P* = .0007) and MLP (*p* ≈ 0) (Table 3). Overall, by applying M2, 121 (LR) and 99 (MLP) patients would have been overtreated, with 57 (LR) and 64 (MLP) patients undertreated (Table 4).

### 3.5 M3, nonimaging variables (scored data)

On LR analysis, the area under the curve dropped slightly to 0.61 (Figure S4A) but remained the same for FIGO and MLP analysis (0.62) (Figure S4D). Calibration analysis revealed the difference between observed and predicted deciles of probability to lie within the 95% limits of agreement (Figure S4B,E). Decision curve analysis showed a small net benefit at a decision probability threshold equivalent to a total FIGO score of 7. This would translate to 5 (LR) and 8 (MLP) patients in 1000 being correctly classified as treatment resistant using the model as opposed to FIGO (Figure S4C,F). Contingency table analysis revealed a significant discordance between the classification of patients using M3 and FIGO, involving 45 patients on LR (McNemar's test *P* = .04) and 50 patients on MLP (*P* = .04) (Table 3). Overall, using this model, 127 (LR) and 206 (MLP) patients would have been overtreated, with 100 (LR) and 73 (MLP) patients undertreated (Table 4).

### 3.6 M4, nonimaging variables (scored and raw data)

M4 generated a slight improvement in area under the curve to 0.63 (LR, Figure S5A) and 0.64 (MLP, Figure S5D). Calibration analysis revealed the difference between observed and predicted deciles of probability to lie within the 95% limits of agreement (Figure S5B,E). At a decision probability threshold equivalent to a total FIGO score of 7, decision curve analysis revealed a small net benefit, which would translate to 5 (LR) and 36 (MLP) patients in 1000 being correctly classified as treatment resistant using the model as opposed to FIGO (Figure S5C,F). Comparing the correspondence between FIGO and M3, 25 patients had a discordant classification on LR (McNemar's test *P* = .27) but 119 patients on MLP (p ≈ 0) (Table 3). M4 would have led to the overtreatment of 136 and 157 patients and undertreatment of 112 and 93 patients, respectively on LR and MLP (Table 4).

### 3.7 M5, imaging variables (scored data)

M5 performed worse than FIGO upon receiver operating characteristic curve analysis on both LR and MLP, with an area under the curve of 0.59 and 0.58, respectively (Figure S6A,D). Calibration analysis revealed the difference between observed and predicted deciles of probability to lie within the 95% limits of agreement (Figure S6B,E). Decision curve analysis showed a small net benefit at a decision probability threshold equivalent to a total FIGO score of 7. This would translate to 10 (LR) and 8 (MLP) patients in 1000 being correctly classified as treatment resistant using the model as opposed to FIGO (Figure S6C,F). There was a significant discordance between FIGO and M5 on LR (76 patients, McNemar's test *p* ≈ 0) but not on MLP (23 patients, *P* = .25) (Table 3). By applying M5, 48 and 111 patients would have been overtreated, with 67 and 79 undertreated cases using LR and MLP, respectively (Table 4).

### 3.8 M6 pretreatment hCG (raw data) + imaging variables (scored data)

Studying receiver operating characteristic curves, FIGO, LR and MLP had an area under the curve of 0.62 (Figure S7A,D) with an uplift in the MLP curve in the bottom left‐hand corner; the area of interest. Calibration analysis revealed the difference between observed and predicted deciles of probability to lie within the 95% limits of agreement (Figure S7B,E). Decision curve analysis revealed a small net benefit at a decision probability threshold equivalent to a total FIGO score of 7. This would translate to 7 (LR) and 38 (MLP) patients in 1000 being correctly classified as treatment resistant using the model as opposed to FIGO (Figure S7C,F). A significant discordance between the classification of FIGO and M6 was observed for both analyses; 40 patients on LR (McNemar's test, *P* = .02) and 89 patients on MLP (*P* = .00014) (Table 3). On LR, 67 patients would have been overtreated and 71 patients undertreated, while on MLP, 135 would have been overtreated and 94 undertreated (Table 4).

## 4 DISCUSSION

Statistical modelling using multivariate LR and a nonlinear counterpart (MLP) yielded only a small improvement over FIGO performance. Six simplified models designed to reduce time and cost demands were explored across a range of performance measures. Conventional descriptors showed the greatest improvement in true positive rate (4.7% on LR and 10.9% on MLP) and positive predictive value (6.7% on LR and 11.7% on MLP). However, this translated to a very small net benefit on decision curve analysis. MLP on M2 generated the largest improvement in area under the curve (0.04) (Figure S3D) and net benefit (48 patients in 1000 being correctly classified as resistant to primary chemotherapy) (Figure S3F). As expected with models intending to improve performance, a significant discordance in classification was widely observed between the models and FIGO. However, risk change analysis revealed that any apparent improvements in FIGO performance arose from the reclassification of primary treatment resistant patients, either from the low‐ to high‐risk group or vice versa. Across M1‐6, 48‐136 patients would have been overtreated by LR and 99‐152 patients by MLP. In addition, 55‐112 patients would have been undertreated by LR and 57‐94 patients by MLP (Table 4).

Our study explored simplified models with the aim to improve ease of use and time‐efficiency, while generating cost‐savings on unnecessary investigations that confer little prognostic value.^37^ It was undertaken for two reasons. First, because of the prevalence of human error within the dataset, which included incorrect, nonsense entries, inaccurate individual scores for each factor and failure to sum the scores correctly. This led to 8.6% of the dataset changing total FIGO score (n = 409) and 2.6% (n = 109 patients) changing risk‐category. This is corroborated in the medical literature, with scored and weighted systems proving especially error prone.^38^ Secondly, literature in other disciplines regarding medical and human‐factors, favour the simplest model to do the job, to minimise error.^39^, ^40^ Concerning the choice of ML model, the MLP is the most popular and widely used for classification, providing adequate flexibility to represent any continuous function.^31^ Although there are numerous alternative models, experience shows that it performs as well as any other on a wide variety of tasks, while there is no a priori means of selecting the best match between task and model.^41^

Clinicians have long acknowledged the imperfections in FIGO, hoping that the system itself could be refined.^2^, ^8^ However, our study has uncovered a fundamental flaw in attempts to improve upon FIGO using data gathered from the target population, because the system itself is used a priori to determine first‐line treatment. Researchers merely have access to a premanipulated dataset which can never be resolved without prior knowledge. The differences in primary treatment between low‐ and high‐risk groups mean that no study can reliably improve the FIGO‐derived prediction of primary single‐agent resistance. Instead, one can only improve the prediction of primary chemotherapy resistance as a whole. While extremely unlikely that high‐risk patients would have responded to single‐agent treatment, a proof of concept has not been performed for ethical purposes. This of course, does not solve the problem concerning high rates of treatment resistance among the low‐risk cohort and is a limitation of the study. Indeed, despite applying LR and MLP techniques to the dataset, only minimal improvements were yielded in FIGO performance, suggesting that the answer lies outside of these eight risk‐factors. Furthermore, regarding clinical implementation, the nonimaging models (M3 and M4) would not be desirable due to the importance of imaging in guiding management decisions. These include determining the length of hospital stay due to the risk of post chemotherapy complications when imaging demonstrates tumour breach of the uterine serosa, or decisions surrounding chemotherapy dosing (eg, methotrexate dose in the presence of cerebral metastases).

Only one previously published study has attempted to improve upon FIGO using univariate and multivariate Cox proportional Hazards model.^42^ However, the primary outcome measure (serological hCG remission and no relapses within the follow‐up period) differs from that of FIGO. Secondary endpoints also differed, including disease progression, relapse or death, hence the results are not comparable. Another study aimed to streamline and match FIGO categorisation rather than improve performance,^20^ while our study used primary treatment response as the predictive model outcome. We consider this approach to be superior because of the known deficiencies in FIGO categorisation and desire to improve upon this system. However, a limitation is that apparent performance improvements actually derive from recategorising patients who are resistant to low‐risk or high‐risk treatment. Among treatment resistant low‐risk patients, this equates to recategorising to the high‐risk group, yet 50% of this cohort will achieve remission with a second‐line single agent^12^, ^27^ and as such, would be overtreated with multiagent chemotherapy. Overtreatment should be avoided, because GTN has a high‐cure rate^11^, ^43^ and most patients are of young, childbearing age. Similarly, changing treatment resistant high‐risk patients to the low‐risk group would lead to considerable undertreatment. Performance improvements must therefore be interpreted with caution. Given the flaws involved with attempts to improve FIGO, the models were not tested upon an independent validation cohort. Further limitations of the study include the inherent bias and difficulties introduced by the retrospective design, for example, missing data, which led to the exclusion of 328 patients (7% of the dataset) across the two centres. Over the study period there have been changes in the clinical management of GTN, including the FIGO scoring system, chemotherapeutic regimens, and advances in radiological imaging modalities. While all these factors cannot be corrected retrospectively, all patients were re‐rescored according to the current FIGO 2000 system prior to analysis. Finally, different hCG assays are used at STDC and CCTDC, which cannot be assumed to be equivalent, despite having the same broad specificity for different hCG isoforms.

Considering the fundamental methodological problems associated with attempts to improve upon FIGO, efforts to enhance the prediction of primary, single‐agent chemotherapy resistance should focus elsewhere. The aim should now be to refine the prediction or even supplement FIGO. Indeed, research is ongoing in this area, evaluating radiological and/or biomarker approaches. An ultrasound‐measured low uterine artery pulsatility index ≤1 is associated with methotrexate resistance among low‐risk patients as a whole^13^ and specifically among those with a total FIGO score of 5‐6, at the highest risk of resistance.^44^ A more recent study has concluded that improved discrimination between patients who are sensitive vs resistant to primary methotrexate can be achieved using a combination of the serum angiogenic factor bone morphogenic protein‐9 (BMP‐9) and uterine artery pulsatility index.^14^ Further studies have focused upon patients with a total score of 5‐6 to refine the FIGO prediction, using hCG cut off levels, a histological diagnosis of choriocarcinoma and the presence of metastatic disease to guide whether these patients should instead receive multiagent therapy first‐line.^12^ Studies focusing upon microRNA biomarkers have, to date, concentrated upon the diagnosis and follow‐up of GTD/GTN,^45^, ^46^, ^47^, ^48^, ^49^ presence of active disease^50^ or progression to GTN,^51^, ^52^, ^53^, ^54^ rather than the prediction of resistance. While research in this field progresses, the immediate strategy should be to streamline FIGO to reduce error, improve efficiency and ease of use.

## AUTHOR CONTRIBUTIONS

Victoria L. Parker, Barry W. Hancock, Allan A. Pacey, Robert F. Harrison and Michael J. Seckl conceived and designed the study. Victoria L. Parker collected and assembled data. Victoria L. Parker and Robert F. Harrison statistically analysed the data. Victoria L. Parker, Matthew C. Winter, John A. Tidy, Naveed Sarwar, Julia E. Palmer, Baljeet Kaur, Katie McDonald, Xianne Aguiar, Kamaljit Singh, Nick Unsworth, Imran Jabbar, Barry W. Hancock, Allan A. Pacey, Robert F. Harrison and Michael J. Seckl contributed to data analysis, verification and interpretation. Victoria L. Parker wrote the manuscript, with editorial input from all authors who approved the final version of the manuscript. Robert F. Harrison and Michael J. Seckl had final responsibility to submit for publication. The work reported in the paper has been performed by the authors, unless clearly specified in the text.

## FUNDING INFORMATION

Victoria L. Parker's salary as a Clinical Research Fellow was funded by large grants CA154 and CA184, awarded by Weston Park Cancer Charity, Sheffield, UK. Michael J. Seckl acknowledges support of the Imperial Biomedical Research Centre funded by the National Institute of Health Research (NIHR) and Experimental Cancer Medicine Centre (ECMC) supported by NIHR and Cancer Research UK. The funders had no role in study design, data collection, analysis, interpretation or writing of the report.

## CONFLICT OF INTEREST

The authors declare no potential conflict of interests.

## ETHICS STATEMENT

Participant consent for our study is covered within the following study ethics approval: reference 16/NE/0292, obtained from the Health Research Authority and North East Newcastle and North Tyneside 1 NHS Research Ethics Committee. All included patients consented for their anonymised data to be held upon a database and used for research purposes.

## Supporting information


**Appendix S1**. Supporting information

## Data Availability

The data sets used and/or analysed during the current study are available from the corresponding author on reasonable request.
