# Supplementary material for: PREDICT‐GTN 1: Can we improve the FIGO scoring system in gestational trophoblastic neoplasia?
Source: Int J Cancer. 2022 Dec 3;152(5):986–97. doi: 10.1002/ijc.34352 (PMC10108153; doi:10.1002/ijc.34352)
Supplement: Supplementary file 1 — Appendix S1. Supporting information [file IJC-152-986-s001.pdf]

PREDICT-GTN 1: Can we improve the FIGO scoring system in Gestational Trophoblastic Neoplasia?

Victoria L Parker, Matthew C Winter, John A Tidy, Barry W Hancock, Julia E Palmer, Naveed Sarwar, Baljeet Kaur, Katie McDonald, Xianne Aguiar, Kamaljit Singh, Nick Unsworth, Imran Jabbar, Allan A Pacey, Robert F Harrison , Michael J Seckl

Supplementary materials table of contents

Title	Page number
Supplementary Table 1. Contingency table for the STDC and CCTDC, demonstrating FIGO performance in predicting primary chemotherapy resistance. STDC n=931, CCTDC n=3260 (total n=4191).	2
Supplementary Figure 1. Bland-Altman plot for FIGO. Tests confirmed non-significance of the least squares slope, p=0.05.	3
Supplementary Figure 2. Performance of M1: all variables (scored data) using logistic regression analysis (A)(B)(C) and the hold-out dataset using Multi-layer Perceptron (D)(E)(F).	4-5
Supplementary Figure 3. Performance of M2: all variables (raw and scored data) using logistic regression analysis (A)(B)(C) and the hold-out dataset using Multi-layer Perceptron (MLP) (D)(E)(F).	6-7
Supplementary Figure 4. Performance of M3: non-imaging variables (scored data) using logistic regression analysis (A)(B)(C) and the hold-out dataset using Multi-layer Perceptron (D)(E)(F).	8-9
Supplementary Figure 5. Performance of M4: non-imaging variables (raw and scored data) using logistic regression analysis (A)(B)(C) and the hold-out dataset using Multi-layer Perceptron (D)(E)(F).	10-11
Supplementary Figure 6. Performance of M5: imaging variables (scored data) using logistic regression analysis (A)(B)(C) and the hold-out dataset using Multi-layer Perceptron (D)(E)(F).	12-13
Supplementary Figure 7. Performance of M6: imaging variables (scored data) using logistic regression analysis (A)(B)(C) and the hold-out dataset using Multi-layer Perceptron (D)(E)(F).	14-15
Appendix	15

Supplementary tables

Supplementary Table 1. Contingency table for the STDC and CCTDC, demonstrating FIGO performance in predicting primary chemotherapy resistance. STDC n=931, CCTDC n=3260 (total n=4191).

CCTDC, Charing Cross Trophoblastic Disease Centre; CR, Complete response to primary chemotherapy; FIGO, International Federation of Gynecology and Obstetrics scoring system; HR, High-risk; LR, Low-risk; n, Number; NPV, Negative predictive value; PPV, Positive predictive value; Rx, Treatment; STDC, Sheffield Trophoblastic Disease Centre; TR, Resistance to primary chemotherapy.

STDC		Primary Rx Response (n patients)		CCTDC		Primary Rx Response (n patients)	
		**TR**	**CR**			**TR**	**CR**
**FIGO Risk**	**LR**	272	563	**FIGO Risk**	**LR**	970	1,809
	**HR**	35	61		**HR**	221	260
	**Sensitivity**	0.11			**Sensitivity**	0.19	
	**Specificity**	0.90			**Specificity**	0.87	
	**PPV**	0.36			**PPV**	0.46	
	**NPV**	0.67			**NPV**	0.65	

Supplementary figures

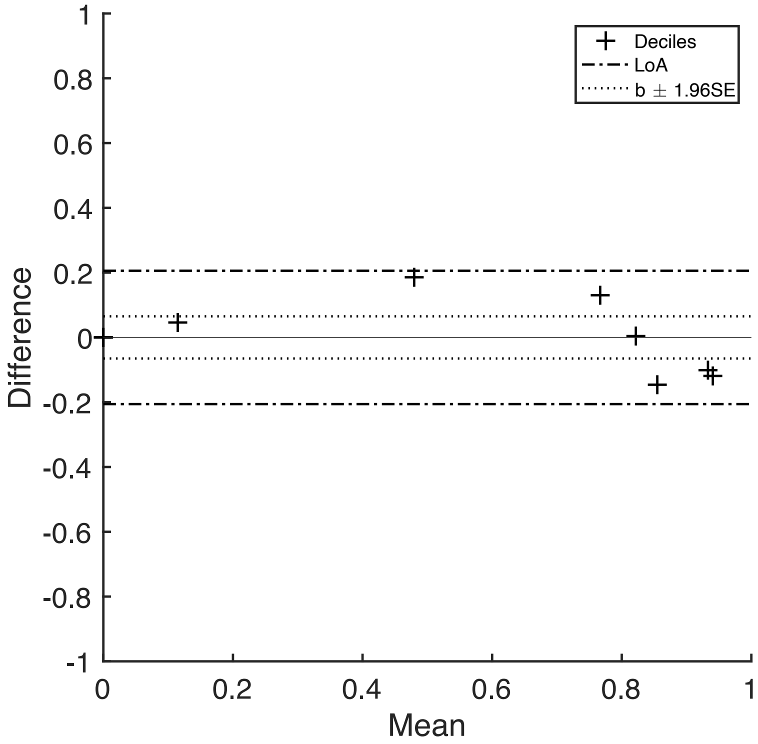

Supplementary Figure 1. Bland-Altman plot for FIGO. Tests confirmed non-significance of the least squares slope, p=0.05.

b, Bias; LoA, 95% limits of agreement; SE, Standard error.

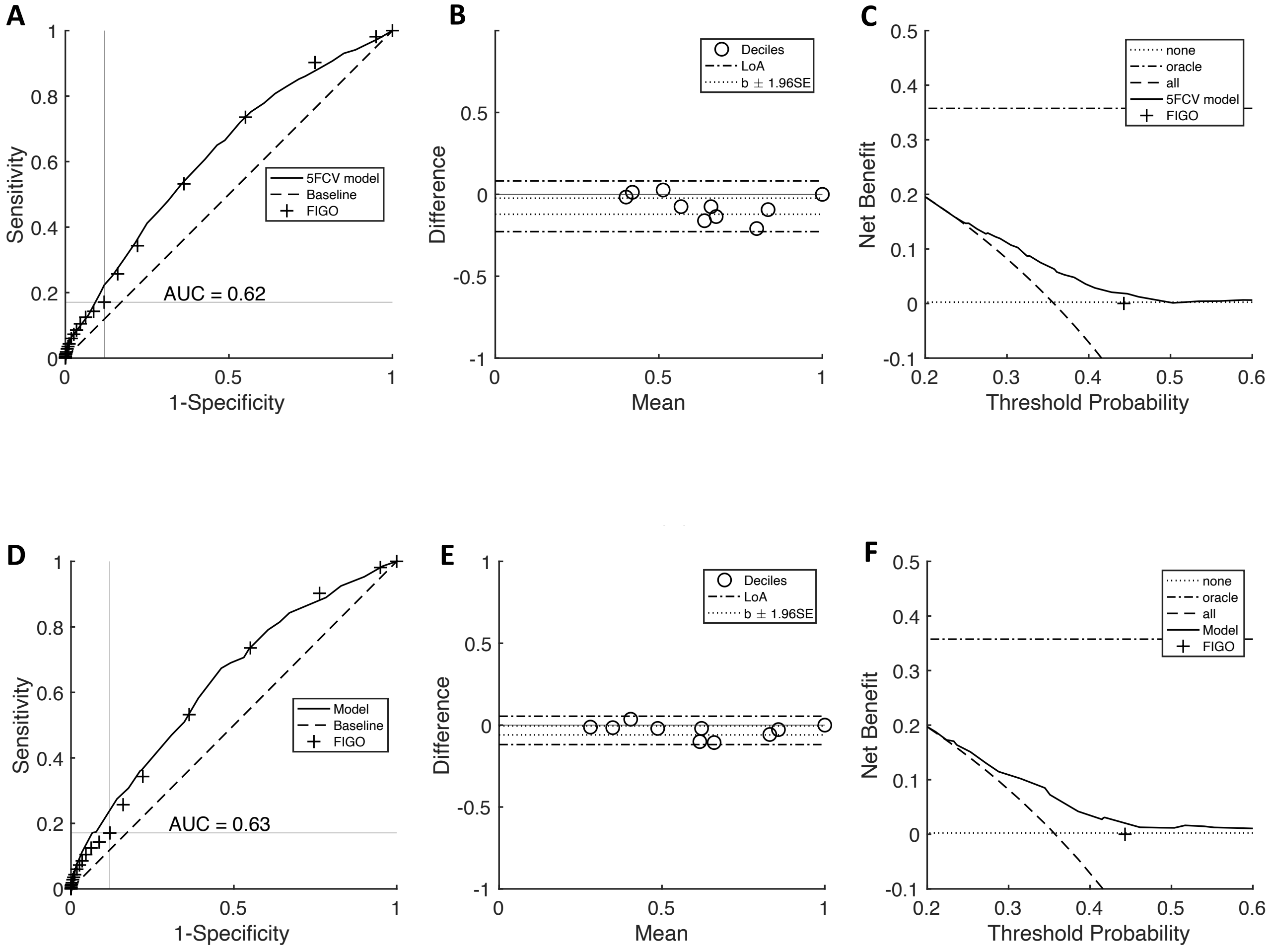

Supplementary Figure 2. Performance of M1: all variables (scored data) using logistic regression analysis (A)(B)(C) and the hold-out dataset using Multi-layer Perceptron (D)(E)(F). (A) and (D) Receiver Operating Characteristics (ROC) Curve comparing M1 with FIGO. (B) and (E) Bland-Altman Calibration plots for M1. Hypothesis tests confirmed non-significance of the least squares slope p=0.05. (C) and (F) Dotted line (treat all patients as low risk (TALR)), the net benefit assuming that no GTN patients will have the outcome (resistance to primary chemotherapy); chained line (oracle), the net benefit associated with a perfect prediction model; dashed line (treat all patients as high-risk (TAHR)), net benefit assuming that all GTN patients will have the outcome; solid line (M1), net benefit when we manage GTN patients

according to the predicted risk of the outcome (primary chemotherapy resistance). + represents the decision probability threshold equivalent to a FIGO score of 7 (decision point between low- and high-risk GTN). Data shown for (A)(B)(C) is the 5FCV result.

AUC, Area under the curve; b, Bias; FIGO, International Federation of Gynecology and Obstetrics scoring system; LoA, 95% limits of agreement; M, Model; SE, Standard error; 5FCV, Five-fold cross-validated.

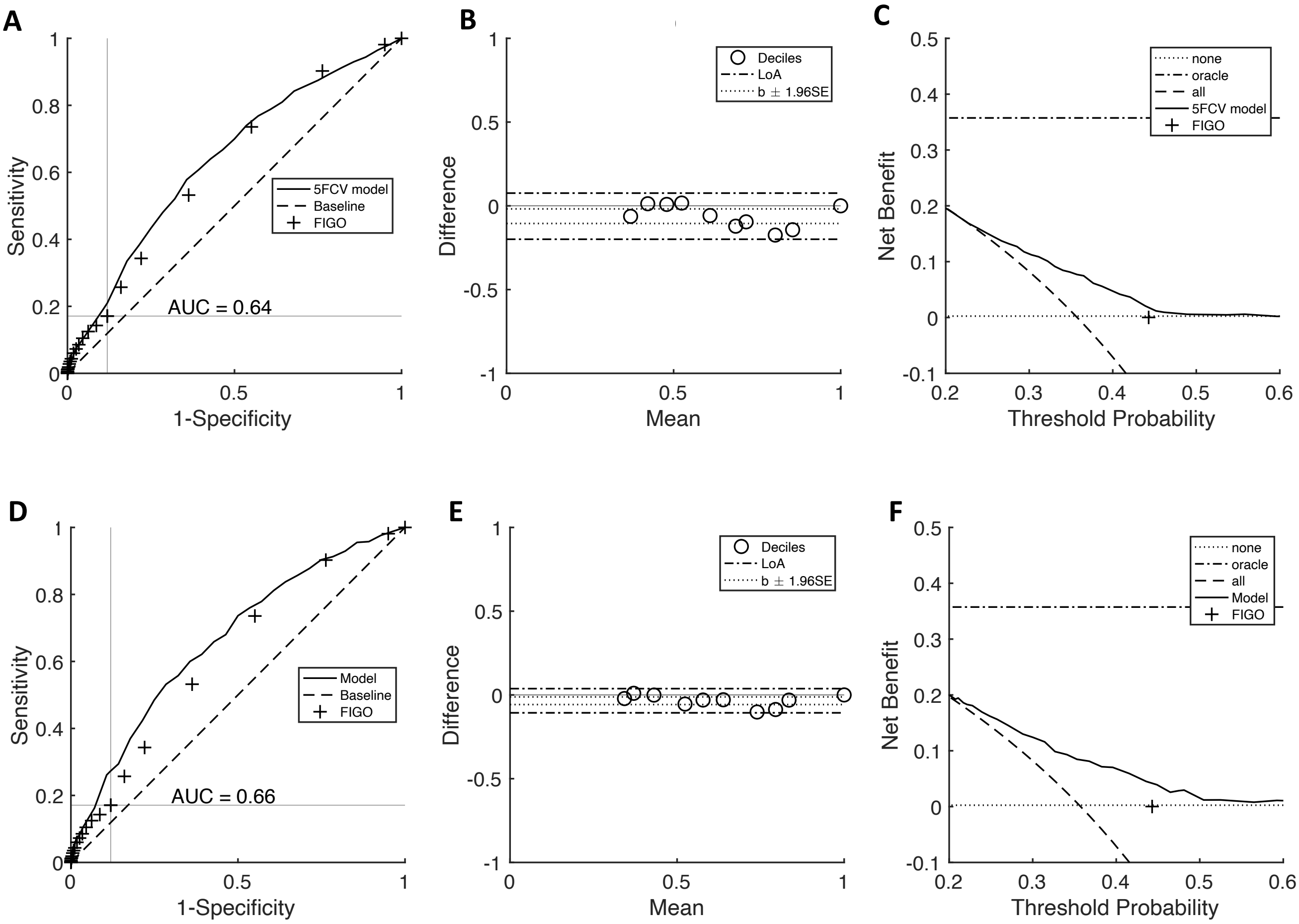

Supplementary Figure 3. Performance of M2: all variables (raw and scored data) using logistic regression analysis (A)(B)(C) and the hold-out dataset using Multi-layer Perceptron (MLP) (D)(E)(F). (A) and (D) Receiver Operating Characteristics (ROC) Curve comparing M2 with FIGO. (B) and (E) Bland-Altman Calibration plots for M2. Hypothesis tests confirmed non-significance of the least squares slope p=0.05. (C) and (F) Decision Curve Analysis (DCA) for M2. Dotted line (treat all patients as low risk (TALR)), the net benefit assuming that no GTN patients will have the outcome (resistance to primary chemotherapy); chained line (oracle), the net benefit associated with a perfect prediction model; dashed line (treat all patients as

high-risk (TAHR)), net benefit assuming that all GTN patients will have the outcome; solid line (M2), net benefit when we manage GTN patients according to the predicted risk of the outcome (primary chemotherapy resistance). + represents the decision probability threshold equivalent to a FIGO score of 7. Data shown for (A)(B)(C) is the 5FCV result.

AUC, Area under the curve; b, Bias; FIGO, International Federation of Gynecology and Obstetrics scoring system; LoA, 95% limits of agreement; M, Model; SE, Standard error; 5FCV, Five-fold cross-validated.

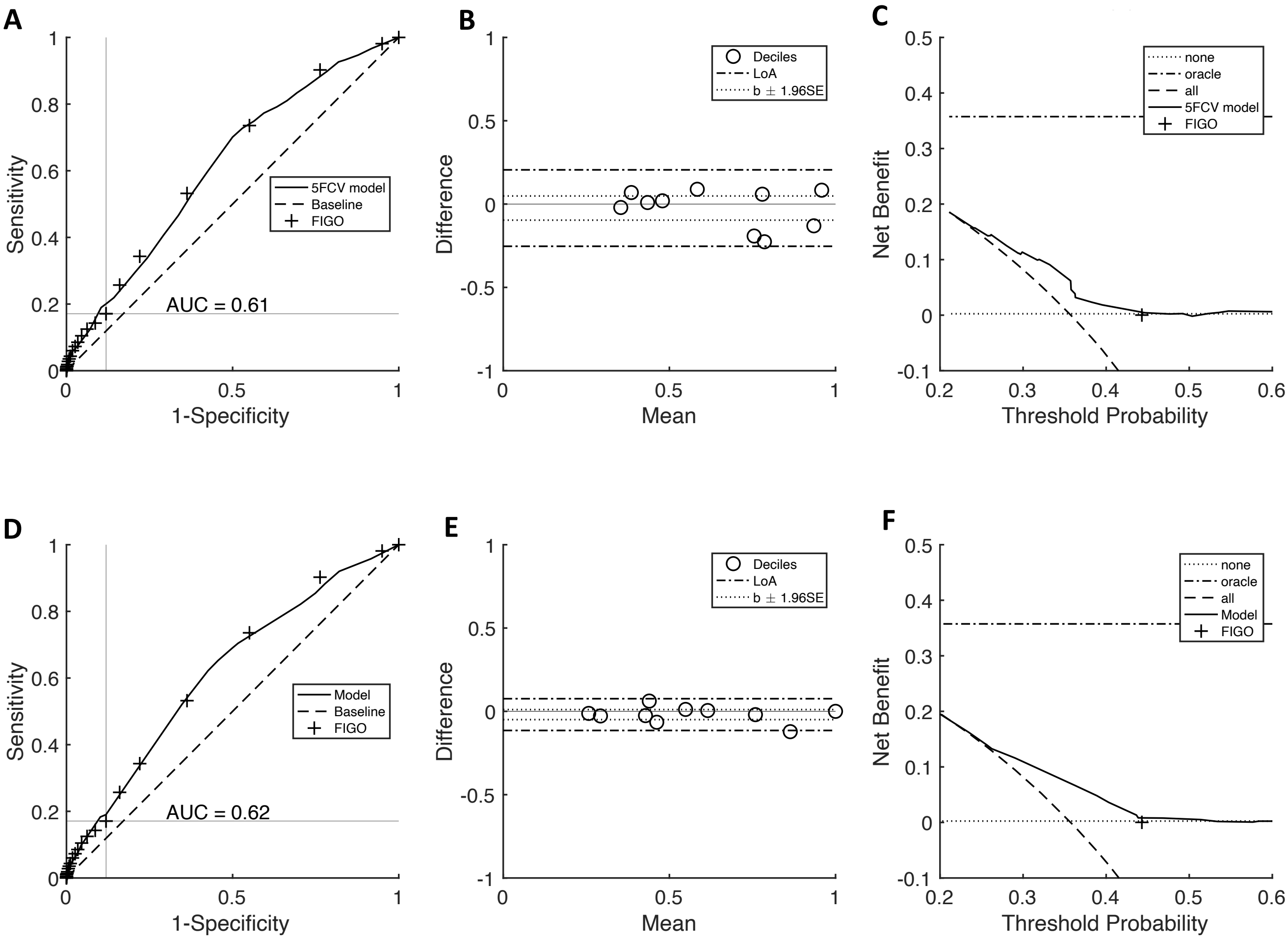

Supplementary Figure 4. Performance of M3: non-imaging variables (scored data) using logistic regression analysis (A)(B)(C) and the hold-out dataset using Multi-layer Perceptron (D)(E)(F). (A) and (D) Receiver Operating Characteristics (ROC) Curve comparing M3 with FIGO. (B) and (E) Bland-Altman Calibration plots for M3. Hypothesis tests confirmed non-significance of the least squares slope p=0.05. (C) and (F) Decision Curve Analysis (DCA) for M3. Dotted line (treat all patients as low risk (TALR)), the net benefit assuming that no GTN patients will have the outcome (resistance to primary chemotherapy); chained line (oracle), the net benefit associated with a perfect prediction model; dashed line (treat all patients as high-risk (TAHR)), net benefit assuming that all GTN patients will have the outcome; solid line (M3), net benefit when we manage GTN patients according to the predicted risk of the outcome (primary

chemotherapy resistance). + represents the decision probability threshold equivalent to a FIGO score of 7. Data shown for (A)(B)(C) is the 5FCV result.

AUC, Area under the curve; b, Bias; FIGO, International Federation of Gynecology and Obstetrics scoring system; LoA, 95% limits of agreement; M, Model; SE, Standard error; 5FCV, Five-fold cross-validated.

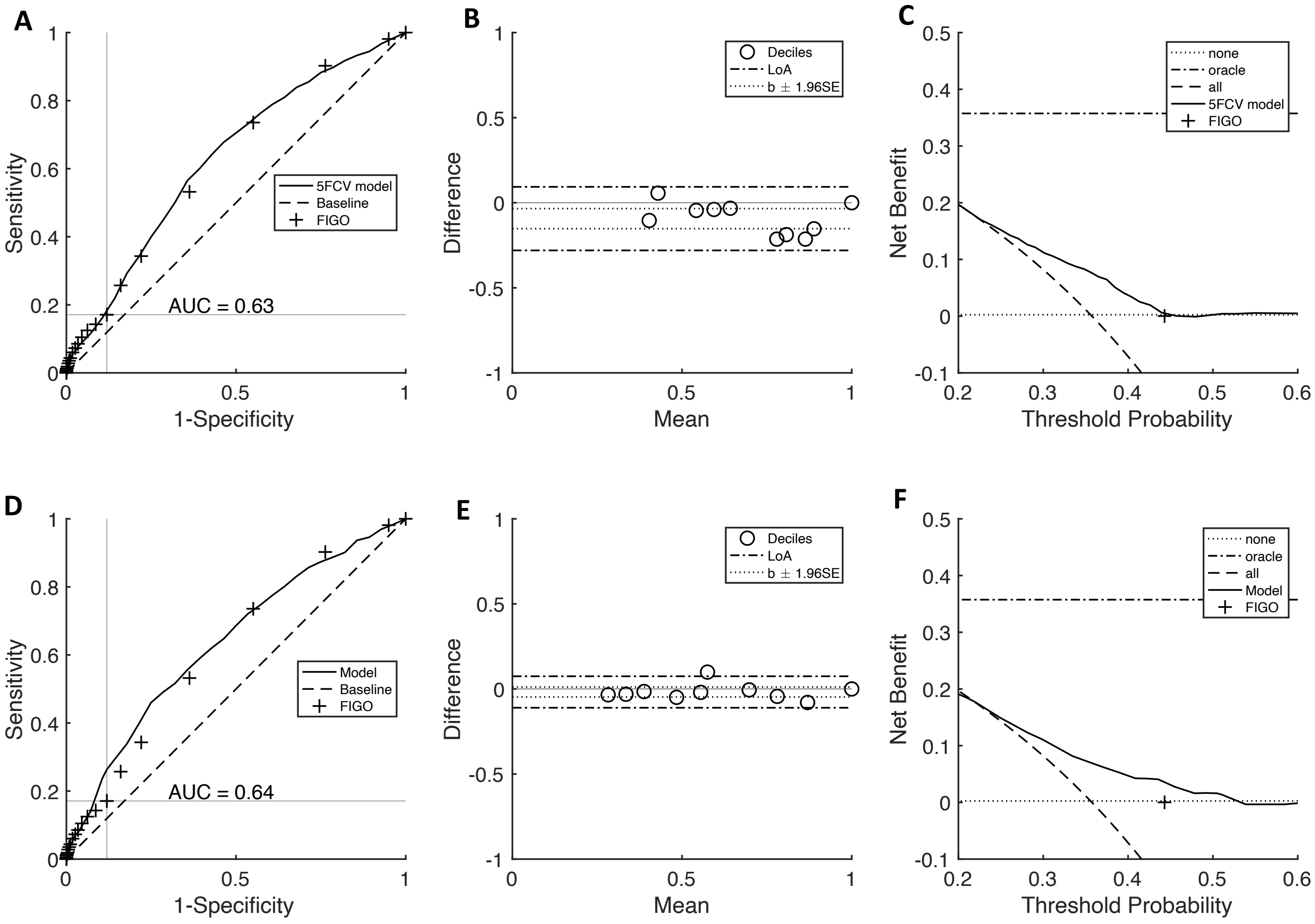

Supplementary Figure 5. Performance of M4: non-imaging variables (raw and scored data) using logistic regression analysis (A)(B)(C) and the hold-out dataset using Multi-layer Perceptron (D)(E)(F). (A) and (D) Receiver Operating Characteristics (ROC) Curve comparing M4 with FIGO. (B) and (E) Bland-Altman Calibration plots for M4. Hypothesis tests confirmed non-significance of the least squares slope p=0.05. (C) and (F) Decision Curve Analysis (DCA) for M4. Dotted line (treat all patients as low risk (TALR)), the net benefit assuming that no GTN patients will have the outcome (resistance to primary chemotherapy); chained line (oracle), the net benefit associated with a perfect prediction model; dashed line (treat all patients as high-risk (TAHR)), net benefit assuming that all GTN patients will have the

outcome; solid line (M4), net benefit when we manage GTN patients according to the predicted risk of the outcome (primary chemotherapy resistance). + represents the decision probability threshold equivalent to a FIGO score of 7. Data shown for (A)(B)(C) is the 5FCV result.

AUC, Area under the curve; b, Bias; FIGO, International Federation of Gynecology and Obstetrics scoring system; LoA, 95% limits of agreement; M, Model; SE, Standard error; 5FCV, Five-fold cross-validated.

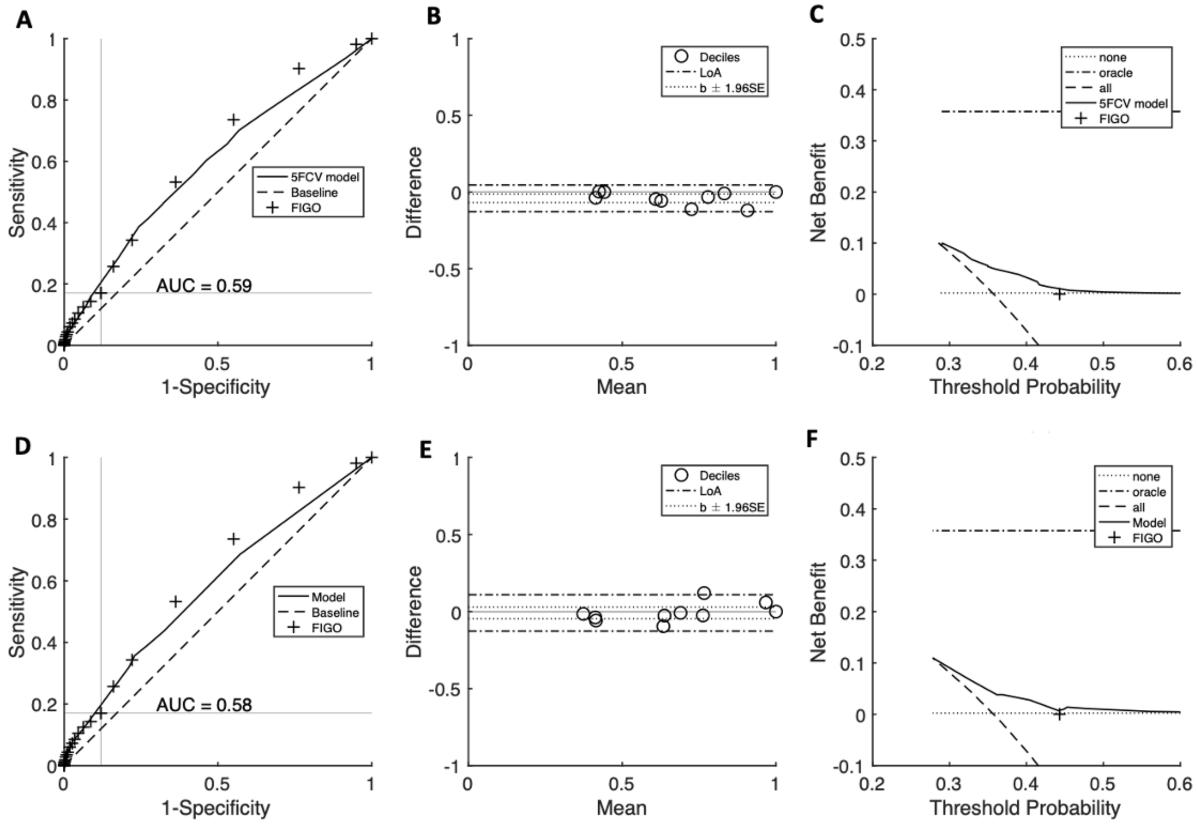

Supplementary Figure 6. Performance of M5: imaging variables (scored data) using logistic regression analysis (A)(B)(C) and the hold-out dataset using Multi-layer Perceptron (D)(E)(F). (A) and (D) Receiver Operating Characteristics (ROC) Curve comparing M5 with FIGO. (B) and (E) Bland-Altman Calibration plots for M5. Hypothesis tests confirmed non-significance of the least squares slope p=0.05. (C) and (F) Decision Curve Analysis (DCA) for M5. Dotted line (treat all patients as low risk (TALR)), the net benefit assuming that no GTN patients will have the outcome (resistance to primary chemotherapy); chained line (oracle), the net benefit associated with a perfect prediction model; dashed line (treat all patients as high-risk (TAHR)), net benefit assuming that all GTN patients will have the outcome; solid line (M5), net benefit when we manage GTN patients according to the predicted risk of the outcome (primary chemotherapy resistance). + represents the decision probability threshold equivalent to a FIGO score of 7. Data shown for (A)(B)(C) is the 5FCV result.

AUC, Area under the curve; b, Bias; FIGO, International Federation of Gynecology and Obstetrics scoring system; LoA, 95% limits of agreement; M, Model; SE, Standard error; 5FCV, Five-fold cross-validated.

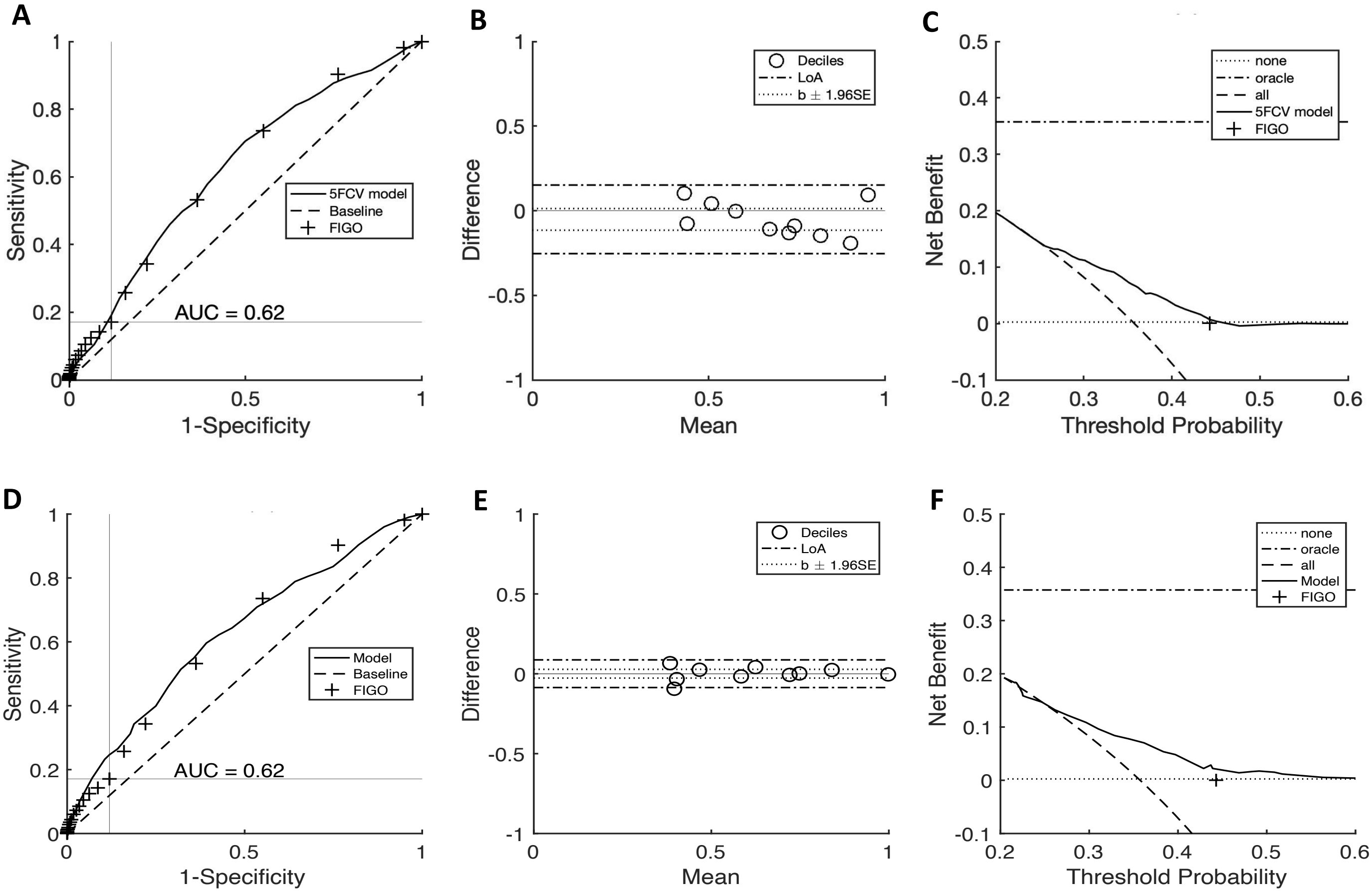

Supplementary Figure 7. Performance of M6: imaging variables (scored data) using logistic regression analysis (A)(B)(C) and the hold-out dataset using Multi-layer Perceptron (D)(E)(F). (A) and (D) Receiver Operating Characteristics (ROC) Curve comparing M6 with FIGO. (B) and (E) Bland-Altman Calibration plots for M6. Hypothesis tests confirmed non-significance of the least squares slope p=0.05. (C) and (F) Decision Curve Analysis (DCA) for M6. Dotted line (treat all patients as low risk (TALR)), the net benefit assuming that no GTN patients will have the outcome (resistance to primary chemotherapy); chained line (oracle), the net benefit associated with a perfect prediction model; dashed line (treat all patients as high-risk (TAHR)), net benefit assuming that all GTN patients will have the outcome; solid line (M6), net benefit when we manage GTN patients according to the predicted risk of the

outcome (primary chemotherapy resistance). + represents the decision probability threshold equivalent to a FIGO score of 7. Data shown for (A)(B)(C) is the 5FCV result.

AUC, Area under the curve; b, Bias; FIGO, International Federation of Gynecology and Obstetrics scoring system; LoA, 95% limits of agreement; M, Model; SE, Standard error; 5FCV, Five-fold cross-validated

Appendix

The Multi-layer Perceptron (MLP) is the most popular and widely used machine-learning model and forms the basis, on a much larger scale, of most of the "deep-learning" algorithms lately becoming prevalent. Here, it is most helpful to think of the MLP as a generalisation of the logistic regression (LR) model. The LR model relates the log-odds of the positive class to the covariates via a weighted sum, i.e., it is a linear function of both the parameters and the covariates. The MLP generalises this to permit an arbitrarily complex, continuous relationship between covariates and log-odds by passing weighted sums of the covariates through successive layers of sigmoidal functions. [31] This ability is known as "universal approximation". [55] The flexibility offered by the MLP introduces technical challenges in the optimisation process, but modern computational solutions are highly developed and reliable. Furthermore, unlike the LR model, the role of MLP parameters is no longer transparent in that the link between change in covariate and change in log-odds is broken. Such models are therefore more suited to prediction than inference.
